# Eicosanoid Profile of Influenza A Virus Infected Pigs

**DOI:** 10.3390/metabo9070130

**Published:** 2019-07-03

**Authors:** Daniel Schultz, Karen Methling, KoInfekt Study Group, Michael Rothe, Michael Lalk

**Affiliations:** 1 Institute of Biochemistry, University of Greifswald, 17487 Greifswald, Germany; 2 Friedrich-Loeffler-Institute, Suedufer 10, 17493 Greifswald, Germany; 3 Lipidomix, 13125 Berlin, Germany

**Keywords:** eicosanoids, lipid mediator, Influenza A virus, infection, pig

## Abstract

Respiratory tract infections caused by the Influenza A virus (IAV) are a worldwide problem for human and animal health. Within this study, we analyzed the impact of IAV infection on the immune-related lipidome (eicosanoids) of the pig as new infection model. For this purpose, we performed HPLC-MS/MS using dynamic multiple reaction monitoring and analyzed lung, spleen, blood plasma and bronchoalveolar lavages. IAV infection leads to collective changes in the levels of the analyzed hydroxyeicosatrienoic acids (HETEs), hydroxydocosahexaenoic acids (HDHAs) and epoxyeicosatrienoic acids (EETs), and moreover, unique eicosanoid changes in several sample types, even under mild infection conditions. In accordance with different mouse infection studies, we observed infection-related patterns for 12-HETE, 15-HETE and 17-HDHA, which seem to be common for IAV infection. Using a long-term approach of 21 days we established an experimental setup that can be used also for bacterial-viral coinfection experiments.

## 1. Introduction

In 2016, lower respiratory tract infections caused more than two millions deaths worldwide [1], with the influenza A virus (IAV) being one of the main etiologic agents. Co-infections of IAV and different bacterial pathogens like *Streptococcus pneumoniae* [2,3] are associated with even higher mortality rates. Humans and pigs are natural hosts of IAV, which was demonstrated during the IAV pandemic in 2009 [4]. Furthermore, the pig can be a mixing vessel [5] for different IAV strains. Compared to other infection model animals like mice, the pig is more related to humans regarding anatomy, physiology and genetics. The organs of pigs and humans are similar in terms of size and function [6,7]. Moreover, there are very close homologies between human and pig protein and genome sequences [8]. The immune system of the pig is more related to the human compared to the mice in terms of immune cell populations [9] and Toll-like receptors [10]. These facts suggest that the pig might be a superior infection model.

Many viruses are able to influence the host metabolic pathways like glycolysis, pentose phosphate pathway, glutaminolysis or fatty acid synthesis to obtain energy for their replication [11]. The IAV is known to be able to change the amount of glycolytic metabolites and nucleotides in cultured animal cells [12]. So far, little is known about the effect on metabolites that are related to the immune system in the context of IAV infection.

Eicosanoids are mainly involved in the activation and resolution of inflammatory reactions [13,14,15,16,17], as well as hypertension [18] and pain [19]. One of the most important precursors is arachidonic acid (AA), which can be converted to prostaglandins (PG) and thromboxanes by the cyclooxygenase pathway (COX). Many of these lipid mediators show pro-inflammatory effects [20]. Hydroxyeicosatrienoic acids (HETEs) produced from AA by lipoxygenases (LOX) are known to be anti-inflammatory, like 12-and 15-HETE, or have pro-inflammatory properties, like 5-HETE [21]. The conversion of AA by cytochrome P450-like enzymes produce epoxyeicosatrienoic acids (EETs) with anti-inflammatory activities [22]. Besides AA, docosahexaenoic acid (DHA), eicosapentaenoic acid (EPA) and linoleic acid are essential precursors for the synthesis of lipid mediators. DHA- and EPA-derived metabolites like hydroxydocosahexaenoic acids (HDHAs) and hydroxyeicosapentaenoic acids (HEPEs) are as well anti-inflammatory eicosanoids and precursors of potent anti-inflammatory mediators namely protectins, maresins or resolvins [15]. Moreover, metabolites derived from linoleic acid like hydroxyoctadecadienoic acids (HODEs) are able to activate G protein coupled receptors [23].

The aim of this study was to analyze the eicosanoid profile of IAV-infected pigs, in order to gain a better understanding of the host immune response. We also investigated which kind of sample material is promising for the analysis of the host immune response to respiratory tract infections on the lipid mediator level. Therefore, samples from the local infection site (lung tissue and bronchoalveolar lavage fluid [BALF]) and samples related to the systemic immune response (blood plasma and spleen tissue) were obtained from the animals and analyzed by HPLC-MS/MS.

## 2. Results

### 2.1. Virus Infection

All animals were tested negative for IAV prior to infection experiment. In contrast, infected animals were tested positive for intranasal virus load (partially tested positive for 2 days post infection [dpi] and all tested positive for 4 dpi, data shown in supplemental material). Furthermore, atelectasis could be observed for the pigs during necropsies, with the exception of three animals at 21 dpi. However, the infected pigs didn’t show clinical symptoms during the trial. Virus detection was negative from 7 dpi for all pigs.

### 2.2. Spleen

**Figure 1:**
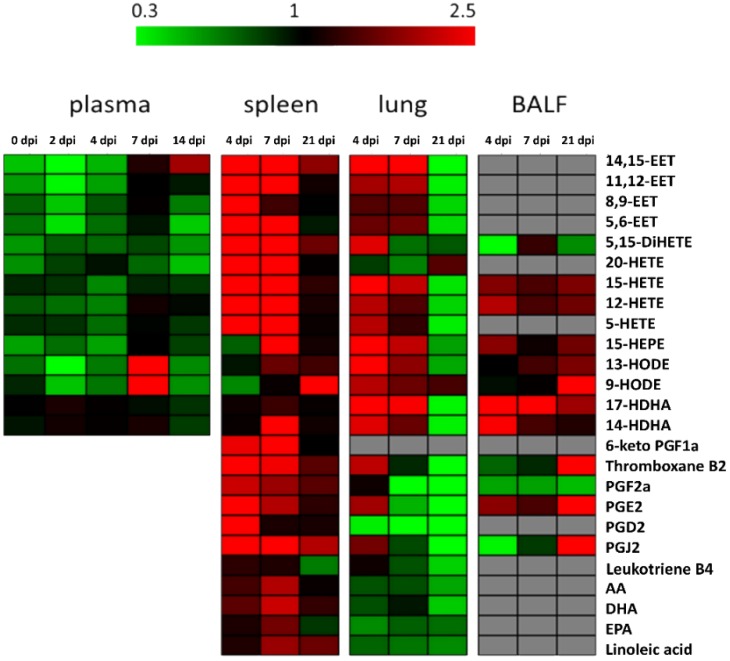
Heatmap displaying fold changes (infection/control) of all measured eicosanoid amounts of all sample types and time points. Decreased levels are shown in green, increased amounts in red and comparable amounts in black. Grey fields: below detection limit.

**Figure 2:**
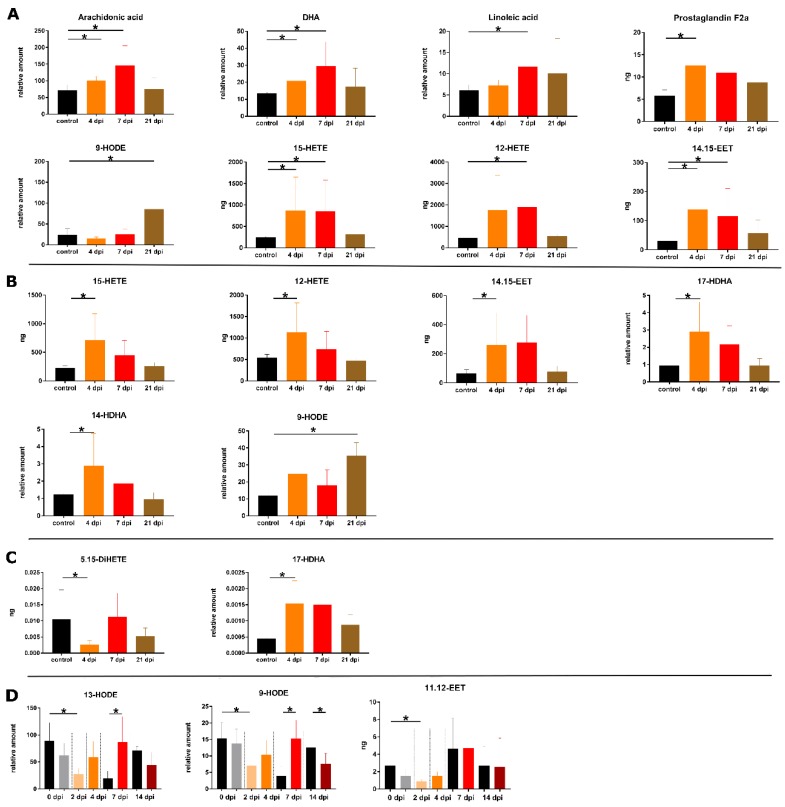
Overview of significant changed amounts of eicosanoids during infection in the spleen (**A**) and the lung (**B**) per g sample; the BALF per 5 mL (**C**) and blood plasma per mL (**D**). Asterisks indicate significant changes (*p*-value ≤ 0.05) using Mann-Whitney *U* test for control (*n* = 3 for tissue and BALF, and n at least 6 for plasma) and infection (*n* = 5 for tissue and BALF, and n at least 6 for plasma). The following color pattern was used: control (black), 4 dpi (orange), 7 dpi (red) and 21 dpi (brown) for **A**–**C**. For plasma (**D**) control samples for all days were illustrated in black and plasma samples from infected animals were shown for 0 dpi (grey), 2 dpi (light orange), 4 dpi (orange), 7 dpi (red) and 14 dpi (dark red).

The spleen tissue showed the most significant changes in the eicosanoid profile during IAV infection compared to all other analyzed sample types (Figure 1). Elevated levels of eicosanoid precursors (AA, DHA and linoleic acid) were detected in the spleen samples of infected animals in particular at 4 and 7 dpi (Figure 2). The observed changes in the eicosanoid precursor levels were exclusively detected in this tissue sample material but not for the lung tissue (Figure 1). Amounts of lipid mediators resulting from AA conversion like pro-inflammatory PGF_2α_, anti-inflammatory 12-and 15-HETE as well as the anti-inflammatory 14,15-EET were found to be increased in the infected animals, in general at 4 and 7 dpi. For the other analyzed EETs, there were also enhanced amounts detected during infection but the differences to controls were not significant. Enhanced amounts of PGF_2a_ were previously reported for patients with chronic inflammatory arthritic diseases [24]. 12-and 15-HETE are able to inhibit the interleukin-6 secretion from macrophages induced by tumor necrosis factor α [25]. EETs are known to have anti-inflammatory effects like the activation of the peroxisome proliferator-activated receptor α (8,9- and 11,12-EET [26]) and suppression of cell degradation by nuclear factor IΚBα (14,15-EET [27]).

For PGF_2a_, a significant response could only be seen at 4 dpi and for 12-HETE only at 7 dpi (Figure 2). At 21 dpi the detected eicosanoid amounts returned to basal levels. Furthermore, the amount of 9-HODE was increased at 21 dpi (Figure 2).

### 2.3. Lung

Various changes in the lipid mediator profile could also be observed for the lung tissue representing local infection sample material (Figure 1). Like for the spleen, elevated amounts of anti-inflammatory 12-and 15-HETE, as well as 14,15-EET could be measured at 4 dpi. 

In addition to the AA-derived lipid mediators, the amounts of 14-and 17-HDHA were enhanced at 4 dpi, which are products of DHA conversion (Figure 2). Perturbations in these HDHAs levels were also found in mice lungs infected with different mouse adapted influenza strains [21,28,29]. For mice, 17-HDHA was shown to enhance specific influenza antibody production [30]. Furthermore, 17-HDHA was able to promote B cell activation and differentiation [30]. With exception of 9-HODE, the analyzed eicosanoids were found in equal amounts to control at 21 dpi (Figure 2). The increased amounts of 9-HODE at 21 dpi were detected like for the spleen. Pro-inflammatory effects of 9-HODE were shown for the calcium mobilization and activation of kinase JNK through the G protein-coupled receptor G2A [23].

### 2.4. BALF

This sample type includes epithelial cells from the lower respiratory tract and immune cells, which is hypothesized to be useful for the analysis of respiratory tract infections. In accordance with the lung analysis, IAV infection led to enhanced amounts of 17-HDHA at 4 and 7 dpi. Moreover, a decrease of 5,15-DiHETE could be observed at 4 dpi for the infected pigs (Figure 2). The lipoxin precursor 5,15-DiHETE is a chemotactic fatty acid for eosinophils [31], which is produced in enhanced amounts from leucocytes after lipopolysaccharide activation [32]. Elevated levels of 5,15-DiHETE are known for patients suffering from chronic inflammation diseases like asthma [33] or rheumatoid arthritis [34]. At 21 dpi, no significant perturbations in the lipid mediator profile of the infected pigs were detected.

### 2.5. Blood Plasma

During early-state IAV infection at 2 dpi, changes in the amount of 11,12-EET were measured. For the following time points (4, 7 and 14 dpi) no significant differences between infection and control animals could be detected. Both, linoleic acid-derived lipids 9-and 13-HODE showed a drop at 2 dpi and increased levels at 7 dpi. In contrast to the pro-inflammatory 9-HODE, the lipid mediator 13-HODE has anti-inflammatory properties as peroxisome proliferator-activated receptor γ agonist [35]. Severe IAV infection in mice showed that the increasing ratio of 13:9-HODE could be useful as a potential biomarker for IAV infection but not for mild infections [29], which is in accordance with our findings. 

## 3. Discussion

### 3.1. Eicosanoid Pattern of IAV-Infected Pigs Compared to Other Animal Infection Models

Most IAV infection studies focusing on eicosanoid analysis used C57BL/6 mice as animal models and analyzed the host lung [28,29]. In the study of Tam et al. [29], also the lipid mediator profile of the mice BALF and human nasal washes were analyzed. The group of Tam used two different IAV strains, the low-pathogenic H3N2 strain X31 and the high-pathogenic H1N1 strain PR8. Both strains are mouse-adapted and led to body weight loss in lethal and sublethal doses between 15% weight loss (X31, sublethal dose) and 25% weight loss (PR8, both doses) of the host [29]. The group of Morita used the high virulent PR8 strain and achieved a host body weight loss of 20% compared to other clinical scores. Furthermore, they used a H1N1 strain (A/California/04/2009) and an avian H5N1 strain (A/Vietnam/1203/04) [28]. Both research groups analyzed the mice lungs at different time points. Tam et al. used for both strains intervals of 2 days starting at 3 dpi until 19 dpi. However, Morita et al. used shorter time intervals starting 6 h after infection and in addition 12 h, 1 dpi and 2 dpi for the PR8 strain infection and for the two other strains 1 dpi and 2 dpi. In our infection experiments, we used 4, 7 and 21 dpi (plus 2 and 14 dpi for plasma) as sampling time points and could not observe body weight loss in the infected pigs. Furthermore, our mild infection led to atelectasis in the lung tissue.

Concerning these different experimental setups, it seems quite difficult to compare the obtained results. Surprisingly, many of the eicosanoid perturbations in the IAV-infected mice are in accordance with these of the infected pigs. For example, in mice infections, changes in the levels of 12-HETE, 15-HETE and 17-HDHA could be observed. We also observed elevated amounts of 12-and 15-HETE (lung, spleen and BALF) and 17-HDHA (lung and BALF) at 4 dpi. Both HETEs, derived from 12-and 15-LOX, have anti-inflammatory effects which may indicate resolution of inflammation even when intranasal virus load is still detectable. This hypothesis is supported by the detection of inhibitory CD8αα expressing T cells as early as 4 dpi in nose and BALF samples in these animals most likely to prevent excessive immune response [36]. Morita et al. showed a very dynamic process of different HETEs and HDHAs under high virulent IAV infection conditions using early sampling time points. Whereas, Tam et al. observed increased amounts of 12-HETE for the X31 strain compared to the PR8 strain at 11 dpi and concluded that some effects may be strain specific for IAV infection. Our experimental setup is more comparable to the work of Tam et al. than to Morita et al. concerning the clinical scoring of the host [28,29]. However, these findings point out that the sampling time point is crucial for the analysis of lipid mediator perturbations. Since we are also interested in bacterial-viral co-infections, we decided to use longer time intervals between the sampling time points that could be necessary for further bacterial and co-infection experiments.

The supposed resolution of inflammation could further be supported by the finding that also the levels of anti-inflammatory 14,15-EET (lung and spleen); 8,9-EET (spleen); 11,12-EET (spleen) and 14-HDHA (lung) were enhanced at 4 dpi. 17-HDHA is known to have a positive effect on host antibody production against IAV [30]. Besides the positive effect on host B cell activation, 17-HDHA is able to inhibit viral nucleoprotein mRNA expression in human lung epithelial cells [28]. The same effect was reported for 12-and 15-HETE [28]. Indeed, our results showed that the infected pigs were able to overcome infection (no virus detection at 7 dpi) and simultaneously most lipid mediator levels returned to basal level. The elevated levels of 9-HODE at 21 dpi detected in lung and spleen were unexpected, and cannot be explained by the experimental settings.

Our measuring method furthermore included Resolvin D5 and Protectin DX [10(S),17(S)-dihydroxy-4Z,7Z,11E,13Z,15E,19Z-docosahexaenoic acid], whose MRM parameters are described in Table S1 in the Supplemental Materials. Both eicosanoids could not be detected in the pig sample material, which is in accordance with Tam et al. for mice and human samples [29]. However, the group of Morita et al. proposed Protectin D1 [10(R),17(S)-dihydroxy-4Z,7Z,11E,13E,15E,19Z-docosahexaenoic acid] to be an important biomarker able to inhibit virus replication, but the commercially available Protectin DX was used as standard compound for analysis and their treatment experiments as previously noted [37].

In general, IAV infection in pigs showed comparable results for eicosanoid analysis to mice infections models with advantages concerning availability of the sample material and similarities to humans in the immunological and physiological parameters. This could be also very helpful for extended research when eicosanoid analysis would be linked to genomic or proteomic research. How bacterial infections and bacterial-viral coinfections will change the lipid mediator response in pigs is unclear, and will be a challenging task for further studies.

### 3.2. Sample Material of Choice To Unravel Changes in the Eicosanoid Profile

Within this study, sample types from local infection (lung tissue and BALF) and systemic immune response (spleen and blood plasma) were analyzed. Changes in the lipid mediator profile could be observed for all analyzed materials. The most changes were detected for lung and spleen. These sample types showed effects for several HETEs, EET and HODEs. Moreover, the spleen showed unique changes in eicosanoid precursor levels, whereas HDHAs perturbations were noticed for lung and BALF, suggesting lung and spleen as an ideal sample material.

Blood plasma and BALF showed only a few significant changes under mild IAV infection conditions. The advantage of plasma is that it can be sampled from the same animal over different time points in required amounts without the need to sacrifice the pig in contrast to the mice infection model. This can be very useful for time dependent analysis in context of activating and resolving effects of eicosanoids. However, the measured eicosanoid plasma levels showed high variabilities. Maybe an enlargement in sample size would be helpful for further experiments. Moreover, using the pig as infection model it is possible to reduce the animal number to obtain enough sample material over the duration of the experiment compared to the mice model. Regarding the high individual response of the pig dealing with IAV infection (standard deviations in Figure 2) it seems useful taking plasma samples of the same animals for the understanding of the individual eicosanoid profile development. We did principal component analysis of plasma eicosanoid levels (data not shown), which showed no clustering for specific animals over time or gender even though sex dependent PG synthesis is known [38]. To handle this, we used 8-week-old castrated piglets and mixed the infection sampling group of a minimum of two male and two female animals.

Regarding further infection studies using respiratory bacterial pathogens and co-infections the authors recommend using lung, spleen and blood plasma as sample material. BALF could be a meaningful addition, if extracellular pathogens play a role for the infection model.

## 4. Materials and Methods 

### 4.1. Chemicals and Standards 

All eicosanoid standard compounds including the deuterated internal standards 12-HETE-d8, 13-HODE-d4, PG E2-d4, Resolvin D1-d5 and AA-d11 were purchased from Cayman chemicals. Solid phase extraction cartridges Bond Elut Certify II (200 mg, 3 mL) were obtained from Agilent^®^. Acetonitrile (99.97%, HPLC-MS grade) was purchased from Th. Geyer^®^, methanol from Roth^®^ and acetic acid (glacial, HPLC grade) from VWR^®^. Butylated hydroxytoluene (≥99.0%) and all other chemicals including hexane, ethyl acetate and sodium hydroxide were obtained from Sigma-Aldrich^®^.

### 4.2. Cells and Virus

Influenza A Virus A/Bayern/74/2009 (H1N1pdm09) was propagated on Madin-Darby canine kidney cells (MDCK II) in MEM containing 0.2% bovine serum albumin, 1 unit/mL penicillin, 1 µg/mL streptomycin and 2 µg/mL N-tosyl-L-phenylalanine chloromethyl ketone (TPCK)-treated trypsin (Sigma-Aldrich). For TCID50 assay, serial tenfold dilutions in infection medium were prepared and added to MDCK II cells on 96-well tissue culture plates. After incubation for three days at 37 °C and 5% CO_2_, each well was monitored for cytopathic effect. Viral titers were calculated according to Spearman-Kärber [39]. Intranasal virus load determination was done using swaps from the nostril and placed in media for incubation as previous reported [40]. The viral titers (TCID_50_) were calculated as described above.

### 4.3. Pig Infection

All animal experiments were approved by the ethics committee of the State Office for Agriculture, Food Safety and Fishery in Mecklenburg-Western Pomerania (LALFF M-V) with the reference number 7221.3-1-035/17. All procedures were carried out in accordance with the relevant guidelines and animal welfare regulations. Animals had free access to water and standard diet.

Eighteen German landrace pigs at four weeks of age were obtained from a commercial high health status herd and separated randomly in three different sheds at the Friedrich-Loeffler-Institut on the island of Riems (Germany) as described previously [40]. Acute influenza virus infection was excluded by matrix gene quantitative RealTime-PCR (AgPath.ID™ One-Step RT-PCR Kit, Applied Biosystems) on nasal swabs (DRYSWAB™, mwe, UK) prior to experimental infection (modified from [41]). Prior to inoculation, blood samples were taken from all animals. Pigs were subsequently infected intranasally by mucosal atomization device (MAD) (Prosys International Ltd, London, UK) with 2 mL H1N1pdm09 on day 0. The control group of three animals received PBS only via the same route. Serum samples were taken on the same days from the same six animals randomly chosen on day 0. Further, necropsy was performed on five animals each on days 4, 7, and 21. Necropsy of control animals was performed on day 30 after first mock-infection. 

From centrifuged EDTA blood, plasma was obtained and directly frozen on dry ice. During necropsy, whole lung, including trachea, was removed to carry out bronchoalveolar lavages (BAL). Briefly, main bronchus of cranial lung lobe was cut with scissors, tube was inserted and 200 mL of sterile PBS supplemented with EDTA was injected with a syringe through the tube into the vessels of the lung lobe, which was then kneaded softly. BALF was removed by syringe. Lung tissue from two different parts of the cranial lung lobe (*pars caudalis* and *pars cranialis*) was excised. The whole spleen was also removed and all different sample types were placed on dry ice immediately after processing.

### 4.4. Eicosanoid Extraction

Completely frozen tissue samples were pulverized using a CP02 automated cryoPREP^®^ (Covaris). The frozen tissue was cut in pieces of approximately 200 mg, transferred in a Covaris tissue tube (extra thick) and cooled down by dipping in liquid nitrogen for 60 s. Then the sample was pulverized with an impact level of four and after cooling, pulverization was repeated once as described above. 50 mg of powder were immediately extracted with 500 µL ice cold methanol containing 0.1% BHT and 500 µL ice cold water. An alkaline hydrolysis step was performed using 300 µL sodium hydroxide (10 mol/L) for 30 min at 60 °C. Immediately after the hydrolysis the pH was adjusted to a value of 6 using acetic acid (10 mol/L). For EDTA plasma samples, an aliquot of 500 µL was used and extracted as described for the tissue material. For BALF extraction, 5 mL BALF was extracted with 5 ml methanol containing 0.1% butylated hydroxytoluene. No alkaline hydrolysis was done for BALF samples. For the analysis, 100 µL of deuterated internal standard mixture (each compound 100 ng/mL in acetonitrile) was added to each sample. Further steps including solid phase extraction were done as previously described [42]. SPE cartridges were conditioned with 3 mL methanol and then with 3 mL sodium acetate buffer (0.1 mol/L sodium acetate, containing 5% methanol [*v*/*v*] at pH value of 6). The samples were loaded and washed with 3 mL 50% methanol. Eicosanoids were eluted using 2 mL of hexane/ethyl acetate (75/25, *v*/*v* containing 1% acetic acid (10 mol/L). Additionally, 50 mg of tissue samples was extracted according to the procedure described above but without hydrolysis with sodium hydroxide for PG measurement.

### 4.5. Eicosanoid Measurement

Extracts were dried under nitrogen flow (TurboVap^®^ from Biotage^®^) and reconstituted in 70 µL 80% acetonitrile or 70 µL 25% acetonitrile (for PG analysis). Dynamic multiple reaction monitoring LC-MS/MS analysis was performed using an Agilent^®^ HPLC system (1200 series), coupled to an Agilent^®^ 6460 Triple quadrupole mass spectrometer with electrospray ionization source in negative mode. The injection volume was 10 µL. The separation was done with a Gemini^®^ NX-C18 column (3 µm, 100 × 2 mm) and equivalent pre column. The mobile phase consisted of A: water with 0.05% acetic acid and B: acetonitrile according to [43] with reduced amount of acetic acid and a flow rate of 0.4 mL/min. For eicosanoid measurement optimized source parameters were: gas temperature 310 °C, gas flow 13 L/min; nebulizer pressure 40 psi; sheath gas temperature 400 °C, sheat gas flow 12 L/min; capillary voltage 3500 V, nozzle voltage 1600 V and delta EMV 400 V. The gradient elution started with 25% B, this was increased within 10 min to 30% B and to 70% B until 15 min. Then, B increased to 100% until 20 min and held for 5 min. After this, the system recovered to starting conditions with a total runtime of 31 min. For precursor analysis of spleen and lung samples, a gradient with a 14 min runtime was chosen starting with 60% B up to 100% B within 5 min. This was held for 3 min and then the system returned to starting conditions. Standard compounds were used for the identification of all detected lipid mediators (retention time, precursor and product ions) and for the optimization of fragmentation parameters (see Table S1 and S2). Calibration curves with MS-certified standards for absolute quantification (range between 0.5 ng/mL and 50 ng/ml for HETEs and EETs; for prostaglandins between 0.25 ng/mL to 50 ng/mL and 5 ng/mL to 1000 ng/mL for precursors, curve type quadratic, weighting 1/x) and deuterated internal standard were used. Eicosanoids classes without appropriate MS-certified standards (HEPE, HODE; HDHA) were normalized to the response of the internal standard and stated as “relative amount” in the plots. Agilent Mass Hunter Qualitative Analysis software and Agilent Mass Hunter Quantitative Analysis software (both version B.07.00) were used for MS data analysis.

### 4.6. Statistics and Data Visualization 

Statistical analysis and data visualization for the samples of control (*n* = 3 pigs) and infected animals (*n* = 5 per time point) was done using GraphPad Prism v. 7.05. For plasma analysis, group size was enlarged (*n* = 6 for control and *n* = 6–8 for infection). Heatmaps were created by MeV v.4.9.0. Tissue material was normalized to the powder weight. Normal distribution of data was tested with Shapiro-Wilk normality test. For significance, Mann-Whitney U-test was performed with a *p* value < 0.05.

## Supplementary Materials

The following are available online at https://www.mdpi.com/2218-1989/9/7/130/s1, Raw data including virus load (excel file); Table S1: MRM parameters for analyzed eicosanoids with qualifier and quantifier ion. And Table S2: MRM parameters for analyzed precursors with qualifier and quantifier ion.
