# Supplementary material for: Eicosanoid Profile of Influenza A Virus Infected Pigs
Source: Metabolites. 2019 Jul 3;9(7):130. doi: 10.3390/metabo9070130 (PMC6680658; doi:10.3390/metabo9070130)
Supplement: Supplementary file 1 [file metabolites-09-00130-s001.zip › Supplementary Materials/Supplementary Tables.pdf]

metabolites

MDPI

Table S1. MRM parameters for analyzed eicosanoids with qualifier[1] and quantifier[2] ion.

compound	precursor ion	product ion	fragmentor [V]	collision energy [V]	cell accelerator voltage [v]	retention time [min]
14,15-EET[1]	319.2	219.1	100	8	4	18.25
14,15-EET[2]	319.2	175.1	100	10	1	18.25
11,12-EET[1]	319.2	179.0	100	8	1	18.50
11,12-EET[2]	319.2	167.2	100	12	2	18.50
8,9-EET[1]	319.2	154.9	100	8	1	18.40
8,9-EET[2]	319.2	151.0	100	10	1	18.40
5,6-EET[1]	319.2	191.0	100	2	1	18.60
5,6-EET[2]	319.2	99.0	100	16	1	18.60
5,15-DiHETE[1]	335.3	255.3	100	12	3	15.50
5,15-DiHETE[2]	335.3	173.2	100	12	6	15.50
20-HETE[1]	319.2	288.9	140	12	2	16.90
20-HETE[2]	319.2	237.0	140	10	3	16.90
15-HETE[1]	319.2	219.2	120	4	4	17.45
15-HETE[2]	319.2	120.9	120	12	3	17.45
12-HETE[1]	319.2	163.0	120	12	1	17.70
12-HETE[2]	319.2	135.2	120	12	3	17.70
5-HETE[1]	319.2	203.3	100	12	5	17.80
13-HODE[1]	295.2	277.2	100	10	2	17.25
13-HODE[2]	295.2	195.1	100	14	1	17.25
9-HODE[1]	295.2	171.0	100	10	1	17.30
9-HODE[2]	295.2	123.1	100	14	3	17.30
17-HDHA[1]	343.2	245.1	100	8	3	17.50
17-HDHA[2]	343.2	201.1	100	10	4	17.50
14-HDHA[1]	343.2	234.1	100	10	4	17.60
14-HDHA[2]	343.2	161.2	100	8	1	17.60
15-HEPE[1]	317.2	255.3	100	4	2	17.00
15-HEPE[2]	317.2	219.2	100	4	4	17.00
Leukotriene B_4[1]	335.2	194.9	120	8	6	15.70
Leukotriene B_4[2]	335.2	58.9	120	6	3	15.70
6-keto PG F_{1a}[1]	369.2	206.8	100	12	4	2.60
6-keto PG F_{1a}[2]	369.2	162.8	100	24	4	2.60
PG D_2[1]	351.3	271.2	80	12	2	11.00
PG D_2[2]	351.3	233.1	80	8	3	11.00
PG E_2[1]	351.3	315.2	80	8	5	9.70
PG E_2[2]	351.3	271.1	80	12	2	9.70
PG J_2[1]	333.2	271.3	80	12	2	14.80
PG J_2[2]	333.2	233.1	80	4	4	14.80
PG F_{2a}[1]	353.2	317.1	100	12	5	8.30
PG F_{2a}[2]	353.2	308.9	100	12	8	8.30
Thromboxane B_2[1]	369.2	195.1	100	6	3	5.90
Thromboxane B_2[2]	369.2	169.0	100	8	6	5.90
Protectin DX[1]	359.2	206.1	100	12	4	15.70
Protectin DX[2]	359.2	153.1	100	12	1	15.70
Resolvin D5[1]	359.2	199.2	80	10	5	15.65
Resolvin D5[2]	359.2	141.0	80	10	2	15.65
12-HETE-d8	327.2	214.0	100	12	4	17.7

13-HODE-d4	299.3	198.2	100	14	6	17.25
PG E2-d4	355.3	275.3	100	12	2	10.00
Resolvin D1-d5	381.2	141.0	100	12	2	13.90

Table S2. MRM parameters for analyzed eicosanoids precursors with qualifier[1] and quantifier[2] ion.

compound	precursor ion	product ion	fragmentor [V]	collision energy [V]	cell accelerator voltage [v]	retention time [min]
AA[1]	303.2	259.1	80	8	2	5.70
AA[2]	303.2	146.9	80	6	1	5.70
EPA[1]	301.2	257.2	100	6	2	5.00
EPA[2]	301.2	203.2	100	10	4	5.00
DHA[1]	327.3	283.2	100	4	2	5.50
DHA[2]	327.3	309.1	100	10	2	5.50
Linoleic acid[1]	279.2	261.3	100	14	7	5.80
Linoleic acid[2]	279.2	234.9	100	12	4	5.80
AA-d11	314.3	270.3	100	10	2	5.70

Abbreviations: hydroxyeicosatrienoic acid (HETE), hydroxydocosahexaenoic acid (HDHA), epoxyeicosatrienoic acid (EET), hydroxyeicosapentaenoic acid (HEPE), hydroxyoctadecadienoic acid (HODE), prostaglandin (PG), arachidonic acid (AA), eicosapentaenoic acid (EPA) and docosahexaenoic acid (DHA).
